# Relating tissue specialization to the differentiation of expression of singleton and duplicate mouse proteins

**DOI:** 10.1186/gb-2006-7-10-r89

**Published:** 2006-10-09

**Authors:** Shiri Freilich, Tim Massingham, Eric Blanc, Leon Goldovsky, Janet M Thornton

**Affiliations:** 1 EMBL-EBI, Wellcome Trust Genome Campus, Hinxton, Cambridge, CB10 1SB, UK

## Abstract

**Background:**

Gene duplications have been hypothesized to be a major factor in enabling the evolution of tissue differentiation. Analyses of the expression profiles of duplicate genes in mammalian tissues have indicated that, with time, the expression patterns of duplicate genes diverge and become more tissue specific. We explored the relationship between duplication events, the time at which they took place, and both the expression breadth of the duplicated genes and the cumulative expression breadth of the gene family to which they belong.

**Results:**

We show that only duplicates that arose through post-multicellularity duplication events show a tendency to become more specifically expressed, whereas such a tendency is not observed for duplicates that arose in a unicellular ancestor. Unlike the narrow expression profile of the duplicated genes, the overall expression of gene families tends to maintain a global expression pattern.

**Conclusion:**

The work presented here supports the view suggested by the subfunctionalization model, namely that expression divergence in different tissues, following gene duplication, promotes the retention of a gene in the genome of multicellular species. The global expression profile of the gene families suggests division of expression between family members, whose expression becomes specialized. Because specialization of expression is coupled with an increased rate of sequence divergence, it can facilitate the evolution of new, tissue-specific functions.

## Abstract 2

An analysis of the relationship between duplication events, the time they took place and the expression breadth of the duplicated genes supports the subfunctionalization model, in which expression divergence following gene duplication promotes the retention of a gene in multicellular species.

## Background

The availability of fully sequenced genomes enables us to explore the links between a species' genotype and its phenotype. For metazoa, one of the most obvious phenotypic characteristics is the appearance of differentiated tissue types. How does the evolution of metazoan genomes relate to tissue differentiation? One aspect of metazoan genome evolution is the emergence of metazoan-specific genes. The contribution of metazoan-specific genes to tissue differentiation was recently demonstrated in a few studies that characterized the tendency of such genes to be specifically expressed in mammalian tissues [1-3]. However, tissue-specific genes are not solely metazoan specific. Pre-metazoan genes (genes that are assumed to have been present in the genome of the unicellular ancestor of animals), despite their general tendency to be globally expressed, are in many cases tissue specific [1]. In some cases in which a pre-metazoan gene is specifically expressed, a duplicate copy of the gene maintains a global expression pattern (for examples, see [4,5]). Gene duplication events therefore provide an additional dimension when studying the relationship between the phyletic age of a gene and its expression breadth. More broadly, gene duplication is a dominant aspect in the evolution of metazoan genomes, and therefore it is important to understand its contribution to tissue differentiation [6,7].

Gene duplication events have been suggested to contribute to the attainment of the complex body organization in metazoan species [6]. A possible mechanism through which gene duplication can contribute to tissue differentiation is described in the recent model of subfunctionalization [8]. According to this model, two daughter genes can accumulate degenerative mutations, resulting in the division of the ancestral function, and hence promote the retention of both duplicate copies in the genome. Division of the expression of the ancestral gene between its daughter duplicates, through the accumulation of mutations in the promoter region, is one mode of function division. Several examples for subfunctionalization of expression were reported for individual genes [7,9,10]. The findings from several studies that used microarray expression information to explore several aspects of the relationship between gene duplication and expression divergence are consistent with these predicted from the subfunctionalization model. Expression divergence between duplicate genes was shown to increase with evolutionary time when studying both temporal (differentiation modes in yeast [11]) and spatial (human tissues [12] and plant tissues [13]) expression divergence patterns, where the divergence of expression occurs relatively shortly after the duplication event. Duplication events of mammalian genes tend to lead toward a tissue-specific expression pattern of the duplicated genes [14].

**Figure 1:**
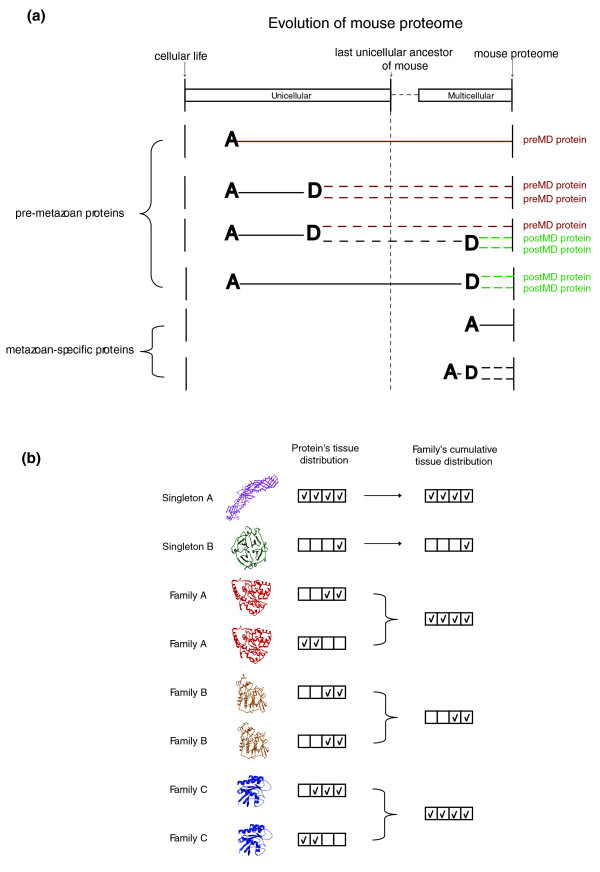
A schematic illustration of concepts described in the text. **(a) **Proteins illustrating different aspects of phyletic age/time of duplication in the mouse proteome, when the calibrated time is the transition from unicellularity to multicellularity. 'A' represents the appearance of a protein in the mouse proteome, and 'D' is a duplication event, leading to the retention of both copies in the mouse proteome. The appearance of a novel protein relates to events where protein contains a novel combination of domains or to events where a protein sequence was changed beyond the recognition of traditional sequence search algorithms, and therefore there is a high likelihood that the protein performs a new function. Pre-metazoan mouse proteins are proteins that have descended from a protein present in the unicellular ancestor of mouse; metazoan-specific proteins are proteins that are unique to the multicellular lineage of metazoa. Because all duplications of metazoan-specific proteins are bound to take place after the transition to multicellularity, proteins from this group are not classified into groups of time of duplication (preMD/postMD). **(b) **Building a cumulative expression profile for protein families. The cumulative expression profile of each family was built by recording all tissues in which at least a single family member is expressed. Singleton proteins, by definition, are single member families and the cumulative distribution is identical to the protein distribution. Family A is an example of complementary expression with no expression overlap between the duplicate proteins; family B is an example of identical expression; and family C is an example of complementary expression with partial expression overlap. The protein cartoons used in this figure are only illustrative. postMD, post-multicellularity duplicates; preMD, pre-multicellularity duplicates.

By using expression information from various mouse tissues, we explore several aspects of the relationship between duplication events and specialization of expression that have not yet been characterized. First, we studied the relationship between duplication events and the expression breadth of the duplicated genes. Previous analysis [14] has shown a general trend toward increased tissue specificity as family size increases. However, because both tissue-specific expression of a gene and the presence of closely related duplicate genes have been independently demonstrated to be associated with a relatively high divergence rate [15-18], we verified that a relationship between expression breadth of a gene and its number of duplicates is not simply derived from the mutual relationship of expression breadth and number of duplicates with the divergence rate. Second, we explored the relationship between duplication events and expression breadth of the duplicated genes within the context of the time when a duplication event took place (Figure 1a). Because spatial expression divergence following gene duplication is more likely in a tissue differentiated environment, duplication events that took place in the unicellular ancestor are likely to affect expression breadth differently than duplication events that took place after the transition to multicellularity. Third, we explored the relationship between duplication events and the cumulative expression breadth of the duplicated gene family (Figure 1b). Finally, in order to illustrate how differentiation of expression contributes to specialization of tissues, we identified and characterized a group of specifically expressed proteins for which no duplicates are detected (singleton proteins). In many cases tissue-specific proteins have duplicates that perform the same function in a larger variety of tissues, but such compensation is less likely for proteins with no such close homologs. The specifically expressed singleton proteins are therefore an ideal group in which to identify and characterize tissue-specific processes.

## Results

### Data

We identified pairs of close homologous mouse proteins by performing an all-against-all BLAST (basic local alignment search tool) search [19] for the entire proteome of mouse (see Materials and methods, below). Here, these protein pairs are termed 'duplicate pairs' because the genes that encode them are likely to have arisen through the duplication of a common ancestor gene. For each protein we counted the number of its duplicate pairs (see Materials and methods, below). Out of 31,535 mouse proteins, we identified 14,384 duplicate proteins (proteins for which at least a single duplicate pair is detected) and 3961 singleton proteins (proteins for which no close homologs are detected). The remaining 13,190 proteins were not classified into either category and were not further analyzed. Duplicate proteins were grouped into 2738 protein families using a single linkage algorithm; specifically, if protein A and protein B form a duplicate pair and protein B and protein C form a duplicate pair, then all three proteins are clustered into the same family.

In order to study whether pre-multicellularity and post-multicellularity duplication events lead to different expression breadth in their resulting duplicates, we studied the relationship between expression breadth and number of duplicate pairs in three groups of mouse proteins (illustrated in Figure 1a): metazoan-specific proteins, post-multicellularity duplicates (postMD), and pre-multicellularity duplicates (preMD). Metazoan-specific proteins are 'novel' proteins that have emerged in a metazoan species (as a result of a domain shuffling [20,21], for example). Because these proteins emerged after the transition to multicellularity, all of the duplication events in their encoding genes must have taken place in a multicellular organism. Pre-metazoan mouse proteins are 'ancient' proteins that have descended from a unicellular ancestor of mouse. Duplication of genes encoding pre-metazoan proteins, unlike duplications of genes encoding metazoan-specific proteins, can either pre-date or post-date the transition to multicellularity. The postMD protein group consists of pre-metazoan mouse proteins that have duplicates that arose through a post-multicellularity gene duplication event. The preMD protein group consists of pre-metazoan mouse proteins that do not have any such 'recent' duplicates, and therefore all of the duplicates of such proteins - if any - have arisen through a pre-multicellularity gene duplication event.

The classification of mouse proteins into the three groups (preMD, postMD, and metazoan specific) was done by comparing the mouse proteins with the proteins of an estimated last unicellular ancestor of mouse. To estimate the proteome of this last unicellular ancestor, we used a combination of the complete protein sequences from a variety of unicellular species (three unicellular eukaryotic species, including fungi and Alveolata, and one bacterial species; see Materials and methods, below). The varied origin of these protein sequences reduces the impact of species-specific gene loss on the classification of preMD/postMD proteins. Proteins with no detectable homologs in any of these unicellular species, or in any other unicellular species out of more than 200 fully sequenced species examined (see Materials and methods, below), were classified as metazoan-specific proteins (*E *value < 10^-3^; similar to the definitions used by Waterston and coworkers [20]). Proteins with detectable homologs were further classified as preMD or postMD using the Inparanoid program [22] (see Materials and methods, below). The Inparanoid clustering procedure identifies orthologs between two species, and allows the identification of duplicates that arose through post-speciation duplication events and duplicates that arose through pre-speciation duplication events. Because the speciation between mouse and its last unicellular ancestor marks the appearance of a multicellular ancestor of animals, the construction of orthologous groups between the proteins of mouse and its last unicellular ancestor enables us to distinguish between pre-multicellularity and post-multicellularity duplicate pairs.

The time gap that exists between the speciation of mouse from its last unicellular ancestor and the speciation from the unicellular species analyzed here suggests that some of the proteins classified as postMD might have been the result of duplication events that took place in a unicellular species. However, the estimated short length of this time gap relative to the period of time since the appearance of a multicellular ancestor of animals [23], together with the major role of duplication events and lineage-specific gene expansions known to be involved in shaping the metazoan gene repertoire, supports the hypothesis that a substantial part of the duplicates of postMD proteins arose in a muticellular ancestor. Therefore, this group is different from the preMD group, in which none of the duplicates of the proteins arose from a post-multicellularity duplication event. In all, 15,394, 3699, and 2231 mouse proteins were classified as metazoan specific, postMD, and preMD proteins, respectively (Table 1).

Expression information was retrieved from 22 adult mouse tissues (Affymetrix U74Av2 GeneChip). Because the sequence similarity between duplicate proteins can lead to promiscuity of their reporting probes, we limited the analysis to probes that uniquely report a single sequence (see Materials and methods, below). In cases in which a probe set is mapped to several splice variants, only the longest transcript is further analyzed. After this filtration the dataset contained expression information for 4914 mouse proteins. For each protein, we recorded its expression breadth according to the absent/present call in each tissue. In order to avoid re-counting similar tissues, the tissues were grouped into 13 clusters and only a single representative member of each cluster was used for analyses comparing expression breadth. All analyses were repeated using an additional microarray expression dataset from mouse tissues (Novartis GNF1M GeneChip) that will be referred to herein as the additional dataset (see Materials and methods, below). The numbers of proteins in the groups analyzed are listed in Table 1 for the main dataset and in (Additional data file 1; Supplementary Table 1) for the additional dataset.

### Expression breadth is negatively correlated with the number of duplicate pairs, independent of the correlation between expression breadth and rate

**Figure 2:**
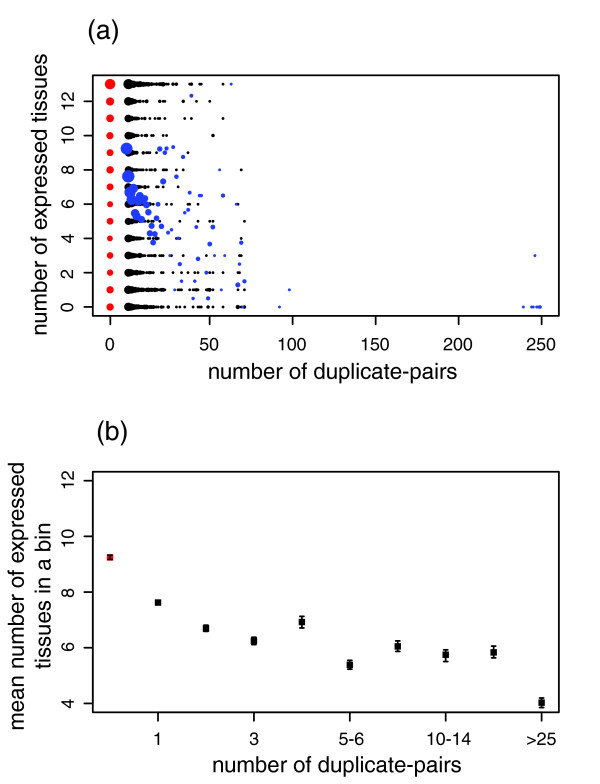
Expression breadth versus the number of duplicate pairs. Red dots indicate singleton proteins, and black dots indicate duplicate proteins. The tissues tested are the 13 cluster-representing tissues. **(a) **The size of the dots represents the number of proteins that have the same number of duplicate pairs and the same expression breadth. The blue dots represent the average expression breadth of proteins with the same number of duplicate pairs. Sample size = 2731 proteins; Kendall's tau = -0.20; *P *value ≤ 2.2 × 10^-16^; 95% confidence interval = -0.22 to -0.17. **(b) **Proteins are ordered according to their number of duplicate pairs and collected into bins of at least 100 proteins. Each point represents a bin. Error bars indicate the standard deviation from the mean, obtained by bootstrapping.

A tendency of mammalian genes from large families to be specifically expressed was previously reported [14]. We tested whether such a tendency exists in our dataset by studying the dependence between expression breadth of a protein and the number of its duplicate pairs. Expression information from the 13 representative tissues was available for 603 singleton proteins and 2128 duplicate proteins (Table 1). A significant negative correlation is observed between the number of duplicate pairs and the expression breadth of a protein (Kendall's tau = -0.20; *P *< 2.2 × 10^-16^). The expression is plotted against the number of duplicate pairs in Figure 2a, which demonstrates the large variation between individual proteins. In order to illustrate the correlation observed for the raw data, we collected the proteins into bins according to the number of their duplicate pairs (small bins were merged with neighbours so that each bin included at least 100 members). When plotting the mean expression in each bin against its ranking order (Figure 2b), one can observe a tendency for proteins with many duplicate pairs to be more specifically expressed. The results are repeatable when using the additional dataset (Additional data file 1; Supplementary Figure 2).

A possible explanation for the dependence between the number of duplicate pairs and the expression breadth is the mutual correlation of both factors with the rate of evolution. Several studies show a correlation between the expression breadth of mammalian proteins and their recent rate of evolution, as inferred by comparison with an ortholog from another mammalian species [15-17]. Other studies report a dependence between recent duplication events and the rate of evolution of the duplicated proteins [18]. For comparison with these studies, we studied the dependencies between rate and expression breadth and between rate and number of duplicates in a subset of 2279 proteins from our dataset that have a rat ortholog (see Materials and methods, below). Compatible with these studies, we found a correlation between rate and expression breadth (Kendall's tau = -0.11; *P *= 1.4 × 10^-14^) and between rate and the number of duplicate pairs (Kendall's tau = -0.06; *P *= 1.5 × 10^-5^). Both correlations are weaker than the correlation reported here between expression breadth and number of duplicates.

We wanted to study whether the relationship between expression breadth and number of duplicates can be explained purely in terms of both factors' mutual correlation with the recent rate of evolution. The first step was to estimate the dependence between expression breadth and number of duplicate pairs using a standard contingency table. Next, the contingency table statistic was compared with those formed by randomizing the data such that only the correlation between the expression and number of duplicate pairs that is due to rate is saved. The randomization was performed by grouping the proteins into bins of a similar recent rate of evolution and shuffling the numbers of duplicate pairs within each group, forming a new set of data that has identical correlation between expression and rate as the original data and that retains a similar correlation between number of duplicate pairs and rate. The contingency table statistic for the original data was compared with those of 10,000 sets of randomized data, and it exceeded them all (test statistic 284, as compared with a maximum of 174 from 10,000 sets of data randomized to be consistent with the null hypothesis). Therefore, there is a relationship between the number of duplicates and the expression breadth that cannot be explained by the mutual correlation with the recent rate of evolution.

### Only post-multicellularity duplication events lead to expression specificity

Our results indicate that, on average, a duplication event leads to a narrower expression profile (Figure 2). Can the narrowing of expression be a factor in promoting the retention of duplicate genes in the genome? If this were true, then only duplication events that took place in a tissue-differentiated environment would be expected to lead to a decrease in the expression breadth of the duplicated genes. In contrast, duplication events that occurred in the unicellular ancestor would not, because retention of the duplicate genes in the genome could not be due to tissue specification.

**Figure 3:**
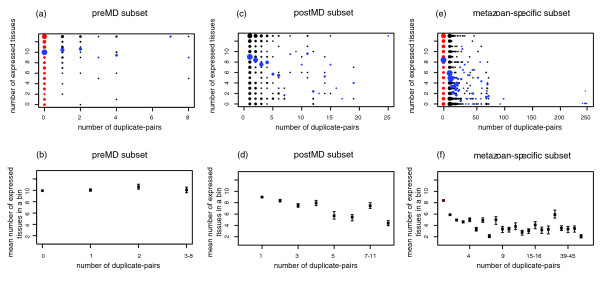
Expression breadth versus the number of duplicate pairs in the preMD, postMD and metazoan-specific subsets. Red dots indicate singleton proteins, and black dots indicate duplicate proteins. The tissues tested are the 13 cluster-representing tissues. **(a,c,e) **The size of the dots represents the number of proteins that have the same number of duplicate pairs and the same expression breadth. The blue dots represent the average expression breadth of proteins with the same number of duplicate pairs. preMD subset (panel a): sample size = 291 proteins, Kendall's tau = 0.001, *P *= 0.51, 95% confidence interval = -0.10 to +0.10); postMD subset (panel c): sample size = 431 proteins, Kendall's tau = -0.15, *P *= 1.2 × 10^-6^, 95% confidence interval = -0.23 to -0.08; metazoan-specific proteins subset (panel e): sample size = 1060 proteins, Kendall's tau = -0.28, *P *= 9.7 × 10^-43^, 95% confidence interval = -0.33 to -0.24. **(b,d,f) **Proteins are ordered according to their number of duplicate pairs and collected into bins of at least 10 proteins. Each point represents a bin. Error bars indicate the standard deviation from the mean, obtained by bootstrapping. postMD, post-multicellularity duplicates; preMD, pre-multicellularity duplicates.

In order to investigate the effect of pre-multicellularity duplication events on expression breadth, we studied the correlation between expression breadth and the number of duplicate pairs in the preMD subset - a subset of mouse proteins whose duplicates have all arisen through pre-multicellularity duplication events. In agreement with the hypothesis, preMD proteins illustrated no tendency toward an increase in tissue specificity accompanying a rise in the number of duplicate pairs (Kendall's tau = 0.001; *P *= 0.51; Figure 3a). When grouping the preMD proteins into bins, the mean expression breadth in all bins remains approximately constant and high (about 10 tissues - the same as for singleton proteins), indicating a global expression for the majority of preMD proteins (Figure 3b).

For comparison with the preMD subset, we studied the correlation between expression breadth and number of duplicate pairs in two subsets of mouse proteins whose duplicates (at least in part) have arisen through a post-multicellularity duplication event, namely the postMD and the metazoan-specific subsets. In agreement with the predicted tendency of post-multicellularity duplication events to lead to tissue-specific expression, we detect a significant negative correlation between expression breadth and number of duplicate pairs in both subsets (postMD subset: Kendall's tau = -0.15, *P *= 1.2 × 10^-6^; metazoan-specific subset: Kendall's tau = -0.28, *P *< 2.2 × 10^-16^). Although the correlations are not high, and although there is a great variability in the distribution of the data (Figure 3c,e), the significance of the correlations indicates that in both datasets there is a tendency of proteins with many homologs to be specifically expressed. This tendency is emphasized when binning the data according to the number of duplicate pairs and plotting the bins against their average expression breadth (Figure 3d,f). The proteins in these two subsets differ in their estimated phyletic age; unlike the 'novel' metazoan-specific mouse proteins, postMD proteins are estimated to be 'ancient' pre-metazoan proteins. The detection of negative correlation in one of the pre-metazoan protein subsets (the postMD subset), together with the inability to detect such correlation in the second pre-metazoan protein subset (the preMD subset), emphasizes the importance of the time of duplication ('D' in Figure 1a), rather than phyletic age ('A' in Figure 1a), in shaping the relationship between duplication events and expression breadth. Such a relationship is only evident in the two subgroups for which duplication events have post-dated the transition to multicellularity.

The postMD and the metazoan-specific subsets differ in the mean expression breadth of bins that have a similar number of duplicate pairs (Figure 3d,f). The average higher expression breadth of the postMD proteins possibly reflects the effect of the phyletic age of a protein on its expression breadth, where metazoan-specific proteins tend to be more tissue specific than pre-metazoan proteins [1].

The findings of this analysis indicate that only duplication events that post-date the transition to multicellularity tend to lead to the development of a tissue-specific expression pattern. This supports the prediction of the subfunctionalization model that tissue specialization is a factor in the retention of duplicate genes in the genome of multicellular organisms. The same analysis was repeated using the additional dataset and the results obtained are compatible with those reported here; only duplicates that have arisen through post-multicellularity duplication events show a tendency to be more specifically expressed (Additional data file 1; Supplementary Figure 3).

### Cumulative tissue distribution of protein families is not correlated with family size

We wished to study the expression breadth of protein families to test whether we can find a correlation not only between expression breadth of an individual protein and its number of duplicate pairs, but also between the size of the family and the cumulative tissue distribution of the entire family (as illustrated in Figure 1b). Two possible scenarios for the relationship between the size and the expression breadth of protein families can account for the tendency of proteins with many homologs to be specifically expressed. The first of these scenarios is complementary expression; a gene duplication event leads to tissue specialization of either one or both daughter genes, yet the two duplicates together cover the expression range of the ancestral gene. The second scenario is identical expression; retention of a duplicate gene in the genome is more likely when its expression is tissue specific. Both duplicate genes will have the same specific expression pattern as the ancestor gene. The olfactory receptor family, one of the largest mammalian protein families, is one example where many members are specifically expressed in one type of cell - the olfactory epithelium [24,25].

We studied the relationship between size and expression breadth in 1249 protein families where expression information was available for at least a single family member. The families vary both in size and in the fraction of the members for which expression information is available. For each protein family a cumulative expression profile was created by summing all tissues in which at least a single family member is expressed (Figure 1b). No negative correlation is observed between the cumulative expression coverage of a protein family and its size (Kendall's tau = -0.03; *P *value for a less one-sided test = 0.02). This is unlike the significant negative correlation observed between the expression breadth of an individual protein and its number of duplicate pairs (Figure 2). Because for many of the families (43%) expression information is available for only a single member, we repeated the analysis using a subset of 189 protein families where expression information is available for at least three-quarters of family members. Again, no negative correlation is observed when using this high coverage subset, and the positive values of both confidence intervals exclude the possibility of negative correlation (Kendall's tau = 0.07; *P *= 1; 95% confidence interval 0.01-0.14). Therefore, although we have only partial expression information for the large majority of families, our data imply that increasing the size of a family does not affect, on average, the cumulative tissue distribution of a family.

**Figure 4:**
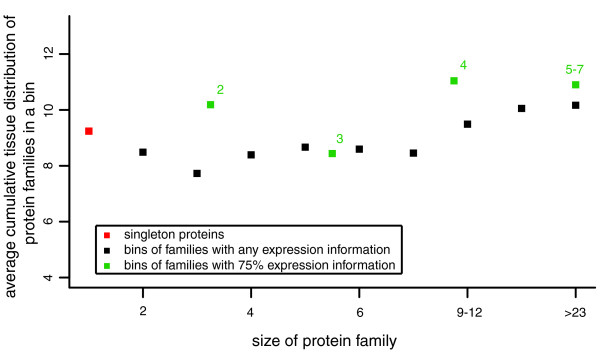
Average cumulative expression coverage in bins of protein families, ordered by size of family. Proteins families for which expression information is available for at least a single member (black) are grouped into bins of at least 35 proteins (total number of families = 1249). Protein families for which expression information is available for at least 75% of the family members (green) are grouped into bins of at least 10 proteins (total number of families = 189). Each point represents a bin. Values on the x-axis describe the size of a family with any expression information (black dots). The size of families with at least 75% expression information (green dots) is the value on top of each green dot.

In Figure 4 we binned protein families according to their size and calculated the average cumulative expression distribution for each bin. As shown in the Figure, the average cumulative distribution of protein families does not decrease when families increase in size and even when using the complete, low coverage dataset (black dots) the average cumulative expression in all bins is approximately identical to the average expression breadth of singleton proteins. A better coverage of family members in the expression data is most likely to strengthen this observation, as indicated by the use of a high coverage subset (green dots). The same observation applies when using the additional dataset (Additional data file 1; Supplementary Figure 4).

Taken together the expression breadth of proteins (Figure 2) with the expression breadth of protein families (Figure 4), our results support the complementary expression model where a duplication event leads to a tissue specialization of one or both copies while the original tissue-distribution of the protein family remains constant.

### Characterisation of singleton proteins whose expression is limited to a few tissues

The unique physiologic role of each mammalian tissue is determined by the unique composition of the genes expressed in the tissue - the tissue's transcriptome. The transcriptome of each tissue comprises genes that are expressed globally and genes whose expression is limited to a subset of tissues [1]. The identification of tissue-specific proteins cannot always shed light on the nature of pathways unique to a tissue, because in many cases tissue-specific proteins have homologs that perform the same function in a larger variety of tissues. Such compensation is less likely for singleton proteins. Singleton tissue-specific proteins are therefore an ideal group for identifying and characterizing tissue-specific processes.

Here, because of the small number of tissue-specific singleton proteins, we analyzed the tissue distribution of proteins whose expression in our dataset is limited to no more than three tissue clusters, which gave us a dataset of 497 duplicate proteins and 60 singleton proteins. Obviously, it is possible that under different conditions, in a different set of tissues (for example, only tissues found in male mice were analyzed here) or during embryonic development, these proteins will be expressed in additional tissues.

From the singleton proteins, 25 proteins are classified as pre-metazoan proteins and 32 proteins are classified as metazoan-specific proteins (three additional proteins did not fall to any phyletic category, as described under Materials and methods, below). The tissue distribution, SWISS-PROT [26] accessions and Gene Ontology (GO) [27] annotations of all 60 tissue-specific singletons are listed in (Additional data file 1; Supplementary Table 2). We looked in detail at a few examples for pre-metazoan and metazoan-specific proteins where annotations are available.

Metazoan-specific proteins are, in many cases, involved in tissue-specific activities [1-3] and their recruitment to the genome therefore accompanies the emergence of highly differentiated organs in multicellular species. Sperm protamine P3, Uteroglobin, Neuromedin U-23 and RAG2, all of which are metazoan-specific proteins whose expression is limited to a few tissues (Additional data file 1; Supplementary Table 2), are examples of proteins for which the function is characteristic of the tissue where they are expressed. Sperm protamine P3, which in our dataset is specifically expressed in the testis, participates in the compaction of chromatin in the spermatid during spermiogenesis [28]. Uteroglobin, which in our dataset is expressed in the lung, is an anti-inflammatory protein expressed in the epithelium cells of pulmonary airways, whose decreased expression is associated with hay fever [29]. Neuromedin U-23 protein, which in our data is expressed in gastrointestinal tract tissues, is thought to stimulate muscle contractions of specific regions in the gastrointestinal tract [30]. The RAG2 protein (V [D]J recombination activating protein 2), which in our dataset is specifically expressed in the thymus, is essential for the assembly of T-cell receptor genes in developing lymphocytes [31].

Tissue-specific expression of pre-metazoan singleton proteins is especially interesting. If we assume that such proteins are integral to a biologic process (being singletons) in the unicellular ancestor, the process must therefore be located in a specialized tissue in multicellular species. It may be that some molecules are only required in one tissue, and therefore the enzymes to make them need only to be expressed there. Alternatively, catabolism of some molecules may be restricted to a single tissue, which acts on behalf of the whole organism. Histidase, homogentisicase, and inositol-oxygenase are examples of specifically expressed enzymes (Additional data file 1; Supplementary Table 2). Histidase catalyses the first step in histidine degradation, a process that takes place in liver and skin of mammals [32]. Homogentisicase participates in the catabolism of tyrosine and phenylalanine, and its expression in mammals is restricted to liver, kidney, small intestine, and prostate [33]. Inositol-oxygenase catalyzes the first committed step in the only pathway of myo-inositol catabolism [34], which in mammals occurs predominantly in the kidney.

In Table 2 we list the tissue distribution of specifically expressed singleton and duplicate genes. The highest fraction of narrowly expressed duplicate proteins (out of the total number of proteins expressed in the tissue) is observed in the brain, whereas the highest fraction of narrowly expressed singleton proteins is observed in the testis, although the sample size is too small to enable a conclusive statistical analysis. One possible explanation for the identification of relatively high number of testis-specific singleton proteins might be the rapid divergence rate of genes that mediate sexual reproduction, a phenomenon that has been suggested to play a role in the establishment of fertilization barriers and speciation [35].

## Discussion

The transition from unicellularity to multicellularity can be viewed as a transition from a studio flat to a 'room-differentiated' house. Some of the essential functions from the studio flat will be found in each room (lamps and doors, for instance). Some functions will become specific to one room type (shower), whereas other functions will have mild adaptations in a few rooms (desk versus dining table). Possibly, some rooms will acquire house-specific functions that cannot be found in the studio flat (conservatory).

Characterization of the equivalent molecular differences underlying tissue diversity sheds light on how the evolution of the genome of multicellular species is related to the appearance of cell types. Duplication of genes was long ago suggested to provide a mechanism for tissue differentiation [6]. Here, we explored several aspects of the differentiation of expression of mammalian genes, especially following duplication events, and tissue specialization.

Several limitations of this analysis must be acknowledged. First, the analysis performed is based only on approximately one-fifth of mouse proteins (proteins that are included in the main expression data), and the expression values are based on a single replicate. However, we were able to repeat the analyses with an additional dataset and obtain compatible results. Second, in both databases the expression data were retrieved from tissues rather than from individual cell types. High coverage, multi-replicate expression data from a wide collection of mammalian cell lines would be ideal for performing such an analysis but is not yet available.

The third limitation is that the sequence similarity search used to estimate the phyletic age of a protein might not be sensitive enough to detect distant homologs of fast evolving proteins because their lesser similarity to distant homologs. Fast evolving pre-metazoan proteins can therefore be misclassified as metazoan-specific proteins (together with genuine metazoan-specific proteins, such as those formed by domain shuffling [20,21] for example). Although such misclassification of fast evolving proteins is possible, for several reasons it is not likely to affect the results of the analysis performed here. Mainly, dependence between the number of duplicate pairs and expression breadth was detected not only for metazoan-specific proteins but also for a group of conserved proteins that have recognized homologs in distant species (the postMD subgroup). The detection of dependence in the postMD group diminishes the likelihood that the reported dependence in the metazoan-specific group is derived from the misclassification of fast evolving proteins. To further preclude the possibility that biases in the distribution of evolutionary rate affected the analysis, we tested the dependence between expression breadth and the number of duplicate pairs for both the fastest and slowest evolving proteins within the metazoan-specific subset, and found a significant dependence within both of these two subgroups (Additional data file 1). Finally, dependence between expression breadth and number of duplicates, demonstrated for the complete dataset, was shown here (see Results, above) to be independent of the recent rate of evolution.

By analyzing the relationship between the expression breadth of a protein and its number of duplicate pairs, we showed that proteins have a tendency to become more specifically expressed after their encoding genes are duplicated. Such a tendency is not observed for the subset of proteins whose duplicates arose through events that pre-date the transition to multicellularity. Therefore, our analysis supports the view that expression divergence, following gene duplication, acts as a stabilizing factor to retain a duplicate gene in the genome of multicellular species. The fact that we do not observe tissue specification of duplicates from pre-multicellularity duplication events suggests that these proteins had undergone a different type of subfunctionalization, such as specialization of their temporal expression or biochemical functions. Unlike the tendency toward specific expression of their protein members, protein families tend to maintain a global expression pattern, therefore implying that the specification of expression between family members is complementary. The findings of this large scale analysis are consistent with the predictions of the subfunctionalization model, which states that the division of the expression (among other functions) of an ancestor gene between its daughter duplicates promotes the retention of a gene in the genome [8]. However, given the lack of information about the expression pattern of a pre-duplication ancestor gene, the analysis performed here can only provide evidence for the current complementary expression between family members, and it does not illuminate the expression pattern of the ancestral state. Other studies have indicated that the expression of duplicate genes is labile and often not consistent with the ancestral state [14,36].

How does the specification of expression lead to the evolution of new, tissue-specific functions? Several lines of evidence indicate that specifically expressed genes diverge at higher rates [15-17], possibly because of less strict functional constraints [37]. As sequence divergence cannot always indicate the functional divergence between duplicate genes, here we focused on a subset of tissue-specific singleton proteins in order to characterize their contribution to the unique physiologic role of the tissue in which they are expressed. We describe a few examples of pre-metazoan and metazoan-specific proteins that participate in such tissue-specific processes. We show examples in which 'metabolic organs', such as kidney and liver, perform functions in mammals that took place in the unspecialized unicellular ancestor, while possibly releasing other mammalian cell types from the constraints involved with performing such functions.

When does the emergence of a new function define a unique role for a tissue? Few reported examples link specification of function to the emergence of new tissue types. One such example is the duplication of an ancestral *opsin *gene into two paralogs: *c-opsin *and *r-opsin*. The paralogs are found, respectively, in the ciliary and rhabdomeric photoreceptor sister cell types, leading to differences in the light sensitivity of those cells. It has been suggested that this duplication event, which took place in an early metazoan ancestor, had allowed the diversification of these two cell types from a precursor photoreceptor ancestor cell [38]. In mammals, ciliary photoreceptor cells have became the main visual photoreceptor cells (rods and cones), whereas rhabdomeric photoreceptor cells are thought to give rise to cells involved in photoperiodicity regulation [38]. Hopefully, the growing number of fully sequenced metazoan species will contribute to our understanding of the way in which the evolution of the metazoan gene repertoire has co-evolved with the development of new tissues.

## Materials and methods

### Expression data

We used microarray data from hybridizations of RNA from mouse tissues to Affymetrix U74Av2 GeneChip using the standard protocol (Affymetrix, Santa Clara, CA, USA). The data are available from ArrayExpress [39] (accession ID = E-HGMP-2). Absence/presence flags were generated using the Microarray Suite 5.0 package (Affymetrix MAS 5.0) with its default settings, as described by Freilich and coworkers [1]. The detection algorithm implemented in MAS5 uses probe pairs intensities to generate a detection call for the transcripts. Each probe pair in a probe set is a factor in determining whether the measured transcript is detected (present), marginal, or not detected (absent). The detection calls are calculated as detailed in the MAS5 manual [40]. We used the default parameters (detection *P *< 0.04) and have treated marginal calls as undetected transcripts. In the text, we refer to this dataset as the main dataset.

As an additional data source, we used Novartis microarray data from hybridizations of RNA from mouse tissues to Novartis GNF1M GeneChip (Novartis. San Diego, California, US) [41]. Absence/presence flags were generated using the Bioconductor implementation of the MAS5 algorithm with its default settings. The data are available on the internet [42]. In the text, we refer to this dataset as the additional dataset. The two datasets are compared in Additional data file 1.

### Construction of tissue clusters

#### Main dataset

The Absence/presence calls from 22 adult male mouse tissues were used to build 13 tissue clusters by constructing a tree and then cutting it into clusters (binary distance measure, average agglomeration method). The tree was cut at a height that allowed maximal variety of tissue clusters but which clustered together highly similar tissues (such as the two testis samples or different parts of the colon). The tissues and the tissue clusters are listed in (Additional data file 1; Supplementary Table 3).

#### Additional dataset

Similarly, the absence/presence calls from 47 adult male mouse tissues were used to build 20 tissue clusters, listed in (Additional data file 1; Supplementary Table 4).

In order to avoid re-counting of similar tissues, we used a single representative of each tissue cluster for analyses, comparing the expression breadth of proteins in mouse tissues. The analyses were repeated using different tissue compositions and compatible results are obtained.

### Identifying nonpromiscuous probe sets and mapping probe sets to mouse proteins

For both chips (main and additional), the individual probes' sequences were aligned against all mouse transcripts predicted in the EnsEmbl [43] (release 30.33 f). The alignment procedure allows a single discrepancy with either the PM (perfect match) or MM (mismatch) sequence. Probes were filtered out if they were not perfectly aligned with any transcript, or they were aligned with more than a single transcript (promiscuous probes). Only probe sets with all probes perfectly matched to a single gene and no matches to any other gene were mapped to proteins. Proteins represented by more than a single probe set were discarded in order to avoid redundancy. In cases for which a probe set is mapped to several splice variants, only the longest transcript is further analyzed. A single and unique probe set therefore represents each of the proteins in our dataset. The main dataset contains expression information for 4914 proteins, and the additional dataset contains the expression information for 13,045 proteins.

### Identification of singleton and duplicate proteins

We conducted an all-against-all BLAST [19] self-search for the entire proteome of mouse (EnsEmbl release 30.33 f). A singleton protein was defined as a protein that did not hit any protein other than itself or its splice variants with *E *value ≤ 0.1, and that recognized itself with an *E *value ≤ 1 × 10^-20^. Proteins that recognize themselves with a high *E *value (possibly as a result of a short sequence or low complexity, which is masked by the SEG filtering subroutine of the BLAST search) will recognize their homologs with a high *E *value. In order not to classify these proteins as singletons, we applied this self-recognition condition.

Two proteins were regarded as duplicates if they met the following criteria: *E *value ≤ 10^-10^; a mutual coverage of 80% between query and hit; and the proteins were not alternative forms encoded by the same gene. For each protein we counted the number of its duplicate pairs (the number of proteins matching these criteria). When counting the number of pairs, all homologs of the protein that are encoded by the same gene (splice variants) are counted only once because they arose through a common gene duplication event. For example, if protein A has three homologues C, D and E, and C and D are splice variants of the same genes, then protein A is considered to have two duplicate pairs. If protein B is a spice variant of protein A, and it also recognizes proteins C, D and E as homologs, then protein B is also considered to have two duplicate pairs. Proteins A and B are not considered to be duplicate pairs of one another.

Only proteins classified as either singletons or duplicates using the strict criteria above were further analyzed.

### Retrieving the evolutionary rate between mouse proteins and their orthologs in rat

Mouse and rat ortholog pairs and their calculated evolutionary rates (dn/ds values) were retrieved from EnsEmbl (downloaded 24 April 2005). Those cases in which a mouse protein matched more than a single rat ortholog were discarded from the analysis, unless one of the orthologous pairs was annotated as BRH (best reciprocal hit). Evolutionary rate (dn/ds) values were obtained for 2279 proteins, including 485 singleton proteins (out of 603) and 1794 duplicate proteins (out of 2128) that are represented in the main expression dataset. The median rate for all pairs is 0.08.

### Assignment of proteins into categories describing their estimated phyletic age

Mouse proteins were classified as pre-metazoan (descendants from a unicellular ancestor of mouse) or metazoan specific, according to the results of a BLAST search [19] against 221 fully sequenced species (including 22 eukaryote species, six of them metazoan). Genomes were downloaded from the COGENT [44] database (version 228). The cut-off used to infer homology was BLAST *E *value < 10^-3 ^(using such a cut-off, no more than one homolog out of 1000 is expected to be a miss-call).

Proteins were only assigned to a single category. The classification process is hierarchical: first proteins with hits to more than ten prokaryote species and/or at least a single hit to a nonmetazoan eukaryote are classified as pre-metazoan (14,957 proteins). Mouse proteins recognizing only metazoan proteins are classified as metazoan specific (15,394 proteins). A total of 266 mouse proteins for which homologs were not inferred in any of the species (including mouse), probably because of short sequence or low complexity, were not included in any of the categories.

### Identification of preMD and postMD proteins

We used the Inparanoid program [22] in order to classify pre-metazoan mouse proteins according to the estimated time (before or after the transition to multicellularity) when their extant duplicates in the mouse proteome arose. Briefly, the Inparanoid program takes as input protein sequence information from two species (A and B) and clusters them into orthologous groups. Each group contains two main orthologs (protein A' from species A and protein B' from species B), which are reciprocal best hits. Proteins from species A that are more similar to A' than to any other protein from species B, and are more similar to A' than A' is similar to B are clustered together with A' in the same orthologous group. These proteins are considered to be in-paralogs of protein A' (proteins that arose through a duplication of the gene encoding protein A' that took place after the speciation of species A from species B). Out-paralogs of protein A' are these proteins that arose through a duplication of the gene encoding protein A' that took place before the speciation of species A from species B. The requirement that in-paralogs of A' are more similar to A' than A' is similar to B' reduces the probability that, as a result of species-specific gene loss, out-paralogs will be classified as in-paralogs.

First, we wished to identify a group of mouse proteins that do not have any duplicates from duplication events that post-date the transition to multicellularity (preMD proteins). Such proteins will therefore not have any in-paralogs when species A is mouse and species B is its last unicellular ancestor. Second, we wanted to identify a group of mouse proteins for which all of their duplicates arose through duplication events that post-date the transition to multicellularity (postMD proteins). Such proteins will therefore have only in-paralogs (but not out-paralogs) when species A is mouse and species B is its last unicellular ancestor. As a reference to the genome of the last unicellular ancestor of mouse, we used the combined sequences of the complete proteomes of *Saccharomyces cerevisiae, Schizosaccharomyces pombe, Escherichia coli*, and *Plasmodium falciparum*.

The combined unicellular protein sequences (downloaded from the Inparanoid database, 20 June 2005), together with the mouse sequences, were used as input for the Inparanoid program. Inparanoid was run with default parameters. The clustering procedure had identified unicellular orthologs for 8305 mouse proteins. Of these, 5886 proteins have mouse in-paralogs and 2419 proteins do not.

The Inparanoid program provides predictions for the in-paralogs that a protein has, but not for its out-paralogs. Here, we used Inparanoid clustering only in order to classify a protein as preMD or postMD, but not in order to count its number of duplicate pairs because duplicates, which are out-paralogs, will not be recognized. Because the classification of protein as preMD or postMD was done separately from the count of its duplicate pairs, we filtered the groups to include only proteins for which all of their duplicate pairs are classified into an Inparanoid orthologous group (not necessarily one group, because some of the duplicate pairs can be out-paralogs). This filtration omits, for example, proteins that have out-paralogs that, as a result of a species-specific gene loss, were not classified to any orthologous cluster. After the filtration the dataset contained 3920 proteins for which the Inparanoid procedure had identified in-paralogs (out of 5886) and 2231 proteins for which no such in-paralogs were identified (out of 2419). For those 2231 proteins without in-paralogs, we can therefore only identify duplicate pairs that have a different unicellular ortholog (those duplicate pairs are predicted to arise through a pre-speciation duplication event). These 2231 proteins are termed 'preMD proteins' here.

From the 3920 proteins with in-paralogs, we filtered out those proteins for which not all of their duplicate pairs are classified into the same Inparanoid orthologous group, leaving 3699 proteins in which all their duplicate pairs are also in-paralogs (they arose through a post-speciation duplication event). These 3699 proteins are termed 'postMD proteins' here.

## Additional data files

The following additional data are available with the online version of this article. Additional data file 1 is a document containing supplementary data in support of the main text (text, tables, and figures).

## Supplementary Material

Additional data file 1 Comparison between the main and the additional dataset, tests of the dependence between expression breadth and the number of duplicate pairs in a group of slow evolving metazoan-specific proteins, and figures and tables as described in the manuscript. Click here for file

## Figures and Tables

**Table 1:** The total number of proteins in the different groups of phyletic age/time of duplication analyzed

	Complete dataset	preMD subset	postMD subset	Metazoan-specific subset
Number of proteins in the mouse proteome	31,535 (4914)	2231 (740)	3699 (618)	15,394 (1915)
Number of proteins that are either singleton or duplicate proteins^a^	18,345 (2731)	811 (291)	2495 (431)	9390 (1060)
Number of singleton proteins	3961 (603)	667 (226)	0 (0)	1960 (792)
Number of duplicate proteins	14,384 (2128)	144 (65)	2495 (431)	7430 (268)

The numbers in parentheses are the numbers of proteins in the group for which expression data were available. ^a^Proteins that did not match the criteria to be either singletons or duplicates were discarded. postMD, post-multicellularity duplicates; preMD, pre-multicellularity duplicates

**Table 2:** Tissue distribution of specifically expressed proteins (expressed in at most three tissue clusters)

Tissue	% Specifically expressed proteins (4573)^a^	Specifically expressed singleton proteins (60)^b^	Specifically expressed duplicate proteins (497)^b^	Phyletic distribution of singleton proteins^c^	
				
				Pre-metazoan (25)	Metazoan- specific (32)
Antrum	0.021 (2958)	2 (0.001)	33 (0.011)	2	0
Appendix	0.007 (2168)	1 (0.000)	7 (0.003)	0	1
Bladder	0.035 (3236)	5 (0.002)	54 (0.017)	3	2
Brain	0.086 (3183)	11 (0.003)	147 (0.046)	4	7
Cecum	0.033 (3219)	7 (0.002)	52 (0.016)	5	2
Distal colon	0.004 (2271)	1 (0.000)	5 (0.002)	0	1
Proximal colon	0.007 (2382)	1 (0.000)	10 (0.004)	0	1
Duodenum	0.045 (3230)	6 (0.002)	83 (0.026)	2	3
Eye	0.066 (3390)	9 (0.003)	121 (0.036)	5	4
Gall bladder	0.040 (3338)	12 (0.004)	70 (0.021)	10	2
Heart	0.014 (2693)	1 (0.000)	23 (0.009)	0	1
Ileum	0.050 (3393)	11 (0.003)	82 (0.024)	6	5
Jejunum	0.043 (3230)	5 (0.002)	72 (0.022)	2	3
Kidney	0.029 (3144)	5 (0.002)	58 (0.018)	4	1
Liver	0.038 (2588)	7 (0.003)	66 (0.026)	5	2
Lung	0.039 (3166)	4 (0.001)	72 (0.023)	0	3
Muscle	0.013 (2241)	2 (0.001)	23 (0.010)	2	0
Spleen	0.065 (3271)	11 (0.003)	81 (0.025)	6	4
Testis	0.074 (3014)	21 (0.007)	90 (0.030)	6	13
Thymus	0.056 (3099)	18 (0.006)	58 (0.019)	10	8
Vas deferens	0.012 (2594)	1 (0.000)	16 (0.006)	0	1

^a^Numbers in brackets: total number of expressed proteins. ^b^Numbers in brackets: fraction out of the total number of proteins expressed in the tissue. ^c^Three of the specifically expressed singleton proteins are not classified to any phyletic category (as described under Materials and methods).

## References

[B1] Freilich S, Massingham T, Bhattacharyya S, Ponsting H, Lyons PA, Freeman TC, Thornton JM (2005). Relationship between the tissue-specificity of mouse gene expression and the evolutionary origin and function of the proteins.. Genome Biol.

[B2] Lehner B, Fraser AG (2004). Protein domains enriched in mammalian tissue-specific or widely expressed genes.. Trends Genet.

[B3] Subramanian S, Kumar S (2004). Gene expression intensity shapes evolutionary rates of the proteins encoded by the vertebrate genome.. Genetics.

[B4] Hendriksen PJ, Hoogerbrugge JW, Baarends WM, de Boer P, Vreeburg JT, Vos EA, van der Lende T, Grootegoed JA (1997). Testis-specific expression of a functional retroposon encoding glucose-6-phosphate dehydrogenase in the mouse.. Genomics.

[B5] Boer PH, Adra CN, Lau YF, McBurney MW (1987). The testis-specific phosphoglycerate kinase gene pgk-2 is a recruited retroposon.. Mol Cell Biol.

[B6] Ohno S (1970). Evolution by Gene Duplication.

[B7] Prince VE, Pickett FB (2002). Splitting pairs: the diverging fates of duplicated genes.. Nat Rev Genet.

[B8] Lynch M, Force A (2000). The probability of duplicate gene preservation by subfunctionalization.. Genetics.

[B9] Adams KL, Cronn R, Percifield R, Wendel JF (2003). Genes duplicated by polyploidy show unequal contributions to the transcriptome and organ-specific reciprocal silencing.. Proc Natl Acad Sci USA.

[B10] Force A, Lynch M, Pickett FB, Amores A, Yan YL, Postlethwait J (1999). Preservation of duplicate genes by complementary, degenerative mutations.. Genetics.

[B11] Gu Z, Nicolae D, Lu HH, Li WH (2002). Rapid divergence in expression between duplicate genes inferred from microarray data.. Trends Genet.

[B12] Makova KD, Li WH (2003). Divergence in the spatial pattern of gene expression between human duplicate genes.. Genome Res.

[B13] Blanc G, Wolfe KH (2004). Functional divergence of duplicated genes formed by polyploidy during *Arabidopsis *evolution.. Plant Cell.

[B14] Huminiecki L, Wolfe KH (2004). Divergence of spatial gene expression profiles following species-specific gene duplications in human and mouse.. Genome Res.

[B15] Duret L, Mouchiroud D (2000). Determinants of substitution rates in mammalian genes: expression pattern affects selection intensity but not mutation rate.. Mol Biol Evol.

[B16] Winter EE, Goodstadt L, Ponting CP (2004). Elevated rates of protein secretion, evolution, and disease among tissue-specific genes.. Genome Res.

[B17] Zhang L, Li WH (2004). Mammalian housekeeping genes evolve more slowly than tissue-specific genes.. Mol Biol Evol.

[B18] Kondrashov FA, Rogozin IB, Wolf YI, Koonin EV (2002). Selection in the evolution of gene duplications.. Genome Biol.

[B19] Altschul SF, Madden TL, Schaffer AA, Zhang J, Zhang Z, Miller W, Lipman DJ (1997). Gapped BLAST and PSI-BLAST: a new generation of protein database search programs.. Nucleic Acids Res.

[B20] Waterston RH, Lindblad-Toh K, Birney E, Rogers J, Abril JF, Agarwal P, Agarwala R, Ainscough R, Alexandersson M, An P (2002). Initial sequencing and comparative analysis of the mouse genome.. Nature.

[B21] Soding J, Lupas AN (2003). More than the sum of their parts: on the evolution of proteins from peptides.. Bioessays.

[B22] O'Brien KP, Remm M, Sonnhammer EL (2005). Inparanoid: a comprehensive database of eukaryotic orthologs.. Nucleic Acids Res.

[B23] Hedges SB, Blair JE, Venturi ML, Shoe JL (2004). A molecular timescale of eukaryote evolution and the rise of complex molecular life.. BMC Evol Biol.

[B24] Young JM, Trask BJ (2002). The sense of smell: genomics of vertebrate odorant receptors.. Hum Mol Genet.

[B25] Hellman A, Chess A (2002). Olfactory axons: a remarkable convergence.. Curr Biol.

[B26] Bairoch A, Apweiler R (2000). The SWISS-PROT protein sequence database and its supplement TrEMBL in 2000.. Nucleic Acids Res.

[B27] Camon E, Magrane M, Barrell D, Binns D, Fleischmann W, Kersey P, Mulder N, Oinn T, Maslen J, Cox A (2003). The Gene Ontology Annotation (GOA) project: implementation of GO in SWISS-PROT, TrEMBL, and InterPro.. Genome Res.

[B28] Aoki VW, Carrell DT (2003). Human protamines and the developing spermatid: their structure, function, expression and relationship with male infertility.. Asian J Androl.

[B29] Benson M, Jansson L, Adner M, Luts A, Uddman R, Cardell LO (2005). Gene profiling reveals decreased expression of uteroglobin and other anti-inflammatory genes in nasal fluid cells from patients with intermittent allergic rhinitis.. Clin Exp Allergy.

[B30] UniProtKB/Swiss-Prot page for entry NEUU_MOUSE. http://us.expasy.org/uniprot/NEUU_MOUSE.

[B31] Agrawal A, Eastman QM, Schatz DG (1998). Transposition mediated by RAG1 and RAG2 and its implications for the evolution of the immune system.. Nature.

[B32] Taylor RG, Levy HL, McInnes RR (1991). Histidase and histidinemia. Clinical and molecular considerations.. Mol Biol Med.

[B33] Granadino B, Beltran-Valero de Bernabe D, Fernandez-Canon JM, Penalva MA, Rodriguez de Cordoba S (1997). The human homogentisate 1,2-dioxygenase (HGO) gene.. Genomics.

[B34] UniProtKB/Swiss-Prot page for entry MIOX_MOUSE. http://ca.expasy.org/uniprot/MIOX_MOUSE.

[B35] Swanson WJ, Vacquier VD (2002). The rapid evolution of reproductive proteins.. Nat Rev Genet.

[B36] Gu Z, Rifkin SA, White KP, Li WH (2004). Duplicate genes increase gene expression diversity within and between species.. Nat Genet.

[B37] Hastings KE (1996). Strong evolutionary conservation of broadly expressed protein isoforms in the troponin I gene family and other vertebrate gene families.. J Mol Evol.

[B38] Arendt D, Tessmar-Raible K, Snyman H, Dorresteijn AW, Wittbrodt J (2004). Ciliary photoreceptors with a vertebrate-type opsin in an invertebrate brain.. Science.

[B39] ArrayExpress at the EBI. http://www.ebi.ac.uk/arrayexpress/.

[B40] Affymetrix. http://www.affymetrix.com/Auth/support/downloads/manuals/data_analysis_fundamentals_manual.pdf.

[B41] Su AI, Wiltshire T, Batalov S, Lapp H, Ching KA, Block D, Zhang J, Soden R, Hayakawa M, Kreiman G (2004). A gene atlas of the mouse and human protein-encoding transcriptomes.. Proc Natl Acad Sci USA.

[B42] GNF Gene Expression Atlas. http://expression.gnf.org.

[B43] Hubbard T, Barker D, Birney E, Cameron G, Chen Y, Clark L, Cox T, Cuff J, Curwen V, Down T (2002). The Ensembl genome database project.. Nucleic Acids Res.

[B44] Janssen P, Enright AJ, Audit B, Cases I, Goldovsky L, Harte N, Kunin V, Ouzounis CA (2003). COmplete GENome Tracking (COGENT): a flexible data environment for computational genomics.. Bioinformatics.

