# Supplementary material for: Relating tissue specialization to the differentiation of expression of singleton and duplicate mouse proteins
Source: Genome Biol. 2006 Oct 9;7(10):R89. doi: 10.1186/gb-2006-7-10-r89 (PMC1794571; doi:10.1186/gb-2006-7-10-r89)
Supplement: Additional data file 1 — Comparison between the main and the additional dataset, tests of the dependence between expression breadth and the number of duplicate pairs in a group of slow evolving metazoan-specific proteins, and figures and tables as described in the manuscript. [file gb-2006-7-10-r89-S1.pdf]

Relating tissue specialisation to the differentiation of expression of singleton and duplicate mouse genes

Shiri Freilich, Tim Massingham, Eric Blanc, Leon Goldovsky, Janet M Thornton

Supplementary data

Comparison between the main and the additional dataset. Two sources of microarray data were analysed for the work reported here: expression information collected on Affymetrix U74Av2 GeneChip arrays and expression information collected on Novartis GNF1M GeneChip arrays (see text). Of the 4914 proteins mapped to a probe-set from the U74Av2 GeneChip, and 13045 proteins mapped to a probe-set from the GNF1M Genechip, there are 3935 proteins in common. We studied the expression pattern (Absence/Presence calls) of these proteins in 14 tissues reported by the two datasets. The number of proteins expressed in each tissue from both sources of data and from the overlap set is shown in Supplementary Figure 1. Most proteins that are reported as present by the GNF1M GeneChip are also reported as present by the U74Av2 GeneChip, but not vice versa. These differences between the GeneChips may be explained by the differences in their sensitivity due to differences in their probe-sets. In the U74Av2 GeneChip most probe-sets are composed of 16 individual reporters, while in GNF1M GeneChip most probe-sets are composed of only 11 individual reporters. Those design differences affect the sensitivity of the MAS5 Presence/Absence call.

Based on the analysis performed and the design of the reporter sets, we believe that the U74Av2 arrays are more sensitive when only reporting absence or presence of expression. Therefore, we chose to report the results from this data source in the paper (main dataset). We have repeated all analyses with the GNF1M arrays (additional dataset).

Testing the dependence between expression breadth and the number of duplicate-pairs in a group of slow-evolving metazoan-specific proteins. In order to verify that the correlation observed in the metazoan-specific protein subset between expression breadth and number of duplicates is not derived from the enrichment of this group in fast-evolving

proteins we have repeated the analysis with a group of slow-evolving metazoan-specific proteins. A significant negative correlation between expression breadth and number of duplicate-pairs were found both in a group of 248 metazoan-specific proteins where mouse-rat evolutionary rate (ka/ks) < 0.05 (group 1) and in a group of 49 proteins where rate < 0.005 (group 2). Correlation was also detected in the group of the 248 fastest evolving proteins (group 3, evolutionary rate > 0.23). The values of evolutionary rates between mouse and rat proteins were obtained as described in the method section in the main text. Correlation for group 1: kendall's tau = -0.28, *P* value = 5.11×10^{-11}. Correlation for group 2: kendall's tau = -0.39, *P* value = 3.27×10^{-5}. Correlation for group 3: kendall's tau = -0.29, *P* value = 6.7×10^{-12}.

Figure legends

Supp. Figure 1: Number of proteins expressed in a tissue, out of 3935 proteins common to the main and additional dataset.

Supp. Figure 2: Analysis based on the additional database. Expression breadth versus the number of duplicate-pairs. Red dots – singleton proteins, black dots – duplicate proteins. The tissues tested are the 20 cluster-representing tissues. (A) The size of the dots represents the number of proteins that have the same number of duplicate-pairs and the same expression breadth. The blue dots represent the average expression breadth of proteins with the same number of duplicate-pairs. Sample size: 7127 proteins; kendall's tau = -0.02; *P* value = 8.7×10^{-4}; 95% confidence intervals: (-0.04, -0.01). (B) Proteins are ordered according to their number of duplicate-pairs and collected into bins of at least 100 proteins. Each point represents a bin. Error bars indicate the standard deviation from the mean obtained by bootstrapping.

Supp. Figure 3: Analysis based on the additional database. Expression breadth versus the number of duplicate-pairs. Red dots – singleton proteins, black dots – duplicate proteins. The tissues tested are the 20 cluster-representing tissues. (A,C,E) The size of the dots represents the number of proteins that have the same number of duplicate-pairs and the same expression breadth. The blue dots represent the average expression breadth of proteins with the same number of duplicate-pairs. preMD subset (A): 489 proteins, kendall's tau = 0.07, *P* value = 0.99, 95% confidence intervals: (-0.01, 0.14); postMD subset (C): 875 proteins, kendall's tau = -0.07, *P* value = 1.5×10^{-3}, 95% confidence intervals: (-0.12, -0.02); Metazoan-specific proteins subset (E): 3281 proteins, kendall's tau = -0.11, *P* value = 3.7×10^{-22}, 95% confidence intervals: (-0.14, -0.09); (B,D,F) Proteins are ordered according to their number of duplicate-pairs and collected into bins of at least 10 proteins. Each point represents a bin. Error bars indicate the standard deviation from the mean obtained by bootstrapping.

Supp. Figure 4: Analysis based on the additional database. Average cumulative expression coverage in bins of protein families, ordered by size of family. Protein families where

expression information is available for at least a single member (black) are grouped into bins of at least 35 proteins (total number of families: 2054). Protein families where expression information is available or at least 75% of the family members (green) are grouped into bins of at least 10 proteins (total number of families: 761). Each point represents a bin. Values on the X-axis describe the size of a family with any expression information (black dots). The size of families with 75% expression information (green dots) is the value on top of each green dot. The tissues tested are the 20 cluster-representing tissues.

Supplement Figure 1

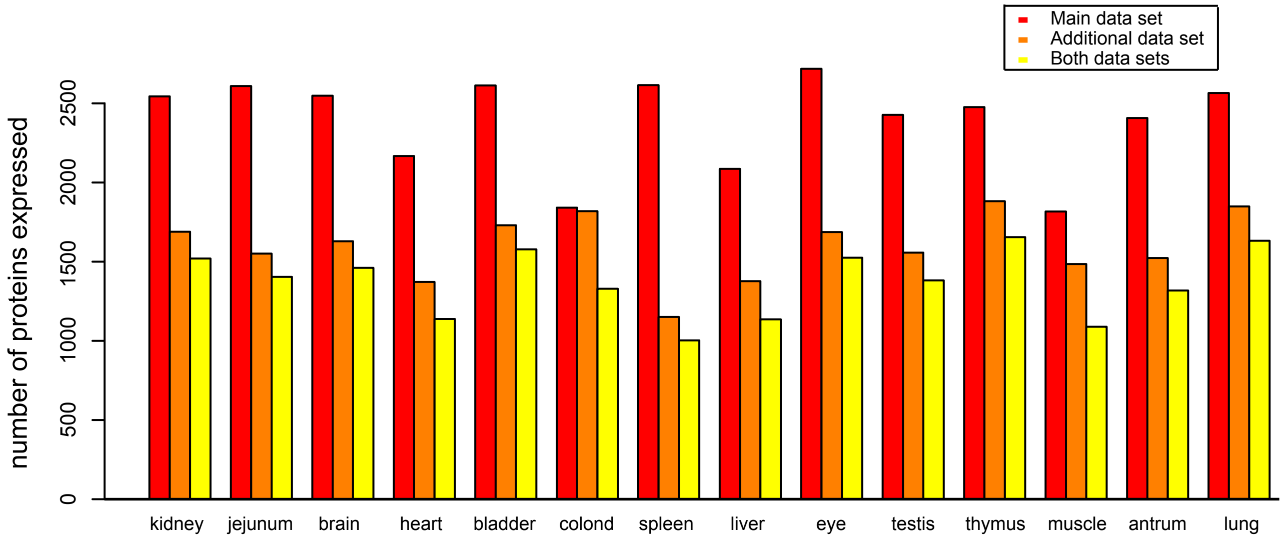

Supplement Figure 2

A

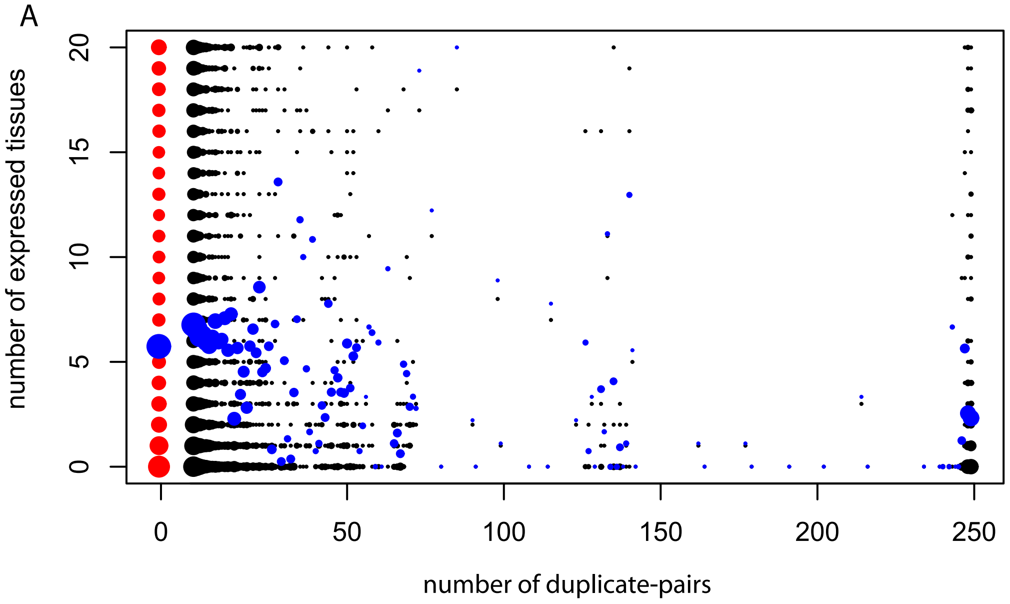

B

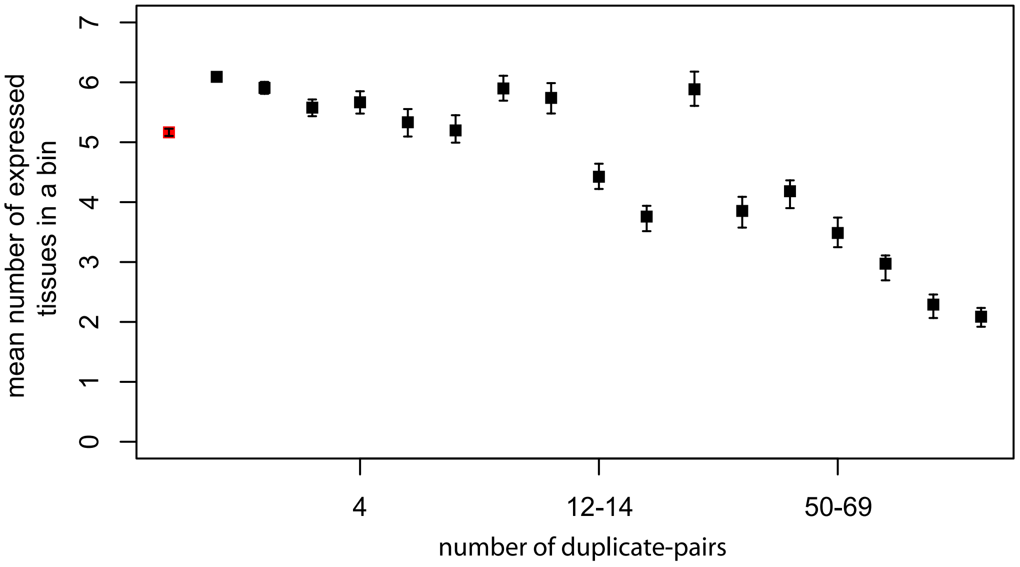

Supplement Figure 3

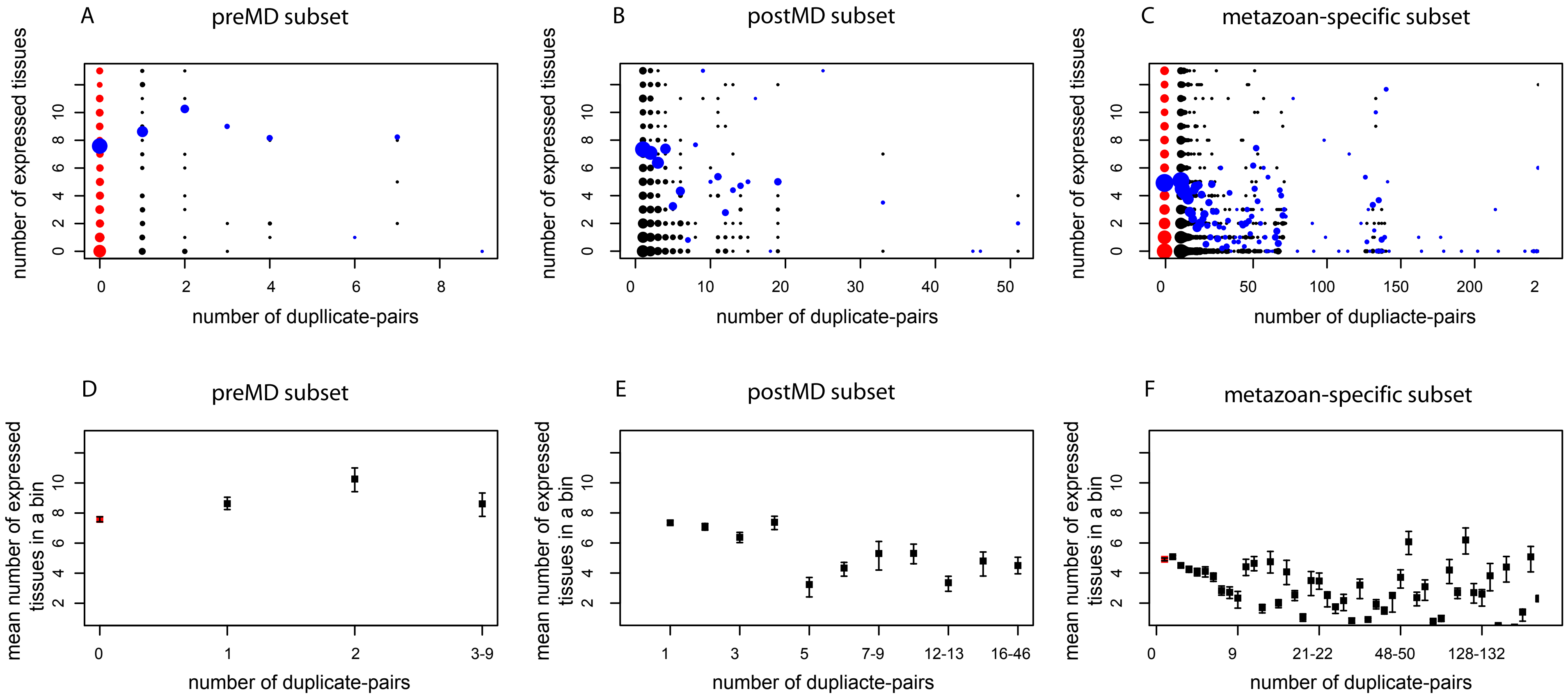

Supplement Figure 4

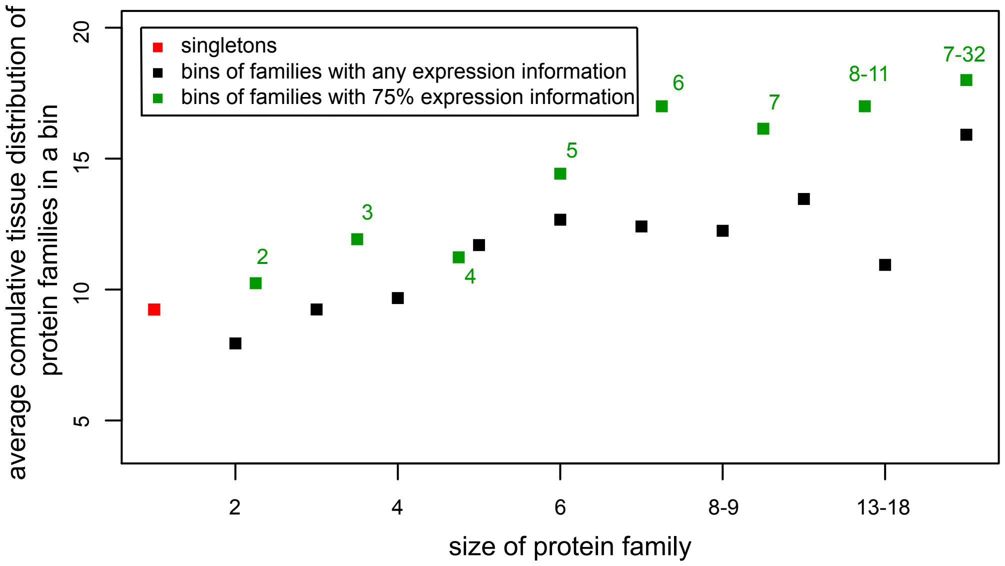

Supplementary Table 1. The total number of proteins in the different groups of phyletic age/time of duplication analysed. The numbers in brackets are the numbers of proteins in the group for which expression data was available in the additional dataset.

	Complete dataset	PreMD subset	PostMD subset	Metazoan-specific subset
Number of proteins in the mouse proteome	31535 (13045)	2231 (1317)	3699 (1335)	15394 (5908)
Number of proteins that are either singleton or duplicate proteins	18075 (7127)	811 (489)	2495 (875)	9390 (3281)
Number of singleton proteins	3691 (2054)	667 (396)	0 (0)	1960 (1041)
Number of duplicate proteins	14384 (5073)	144 (93)	2495 (875)	7430 (2240)

Supplementary Table 2. Annotations of specifically expressed singleton proteins (expressed in at most 3 tissues).

	ENSEMBL id (phyletic group*)	SWISSPROT accession and name	GO Molecular Function	GO Biological Process	GO Cellular Component
antrum	ENSMUSP00000023510 (PM)	PYR5_MOUSE Uridine 5'-monophosphate synthase	nucleobase, nucleoside, nucleotide and nucleic acid metabolism,'de novo' pyrimidine base biosynthesis,aromatic compound metabolism,biosynthesis,nucleobase metabolism,heterocycle metabolism,	transferase activity,lyase activity,	
antrum	ENSMUSP00000047720 (PM)				
appendix	ENSMUSP00000043369 (MS)	IL5_MOUSE Interleukin-5 [Precursor]	phosphorus metabolism,phosphate metabolism,immune response,response to biotic stimulus,positive regulation of metabolism,peptidyl-tyrosine phosphorylation,peptidyl-tyrosine modification,regulation of phosphate metabolism,regulation of metabolism,macromolecule metabolism,positive regulation of phosphate metabolism,positive regulation of peptidyl-tyrosine phosphorylation,	receptor binding,	
bladder	ENSMUSP00000006692 (PM)	ER19_MOUSE Diphosphomevalonate decarboxylase	alcohol metabolism,lipid metabolism,cholesterol biosynthesis,phosphorus metabolism,phosphate metabolism,biosynthesis,macromolecule metabolism,	nucleotide binding,kinase activity,lyase activity,	
bladder	ENSMUSP00000021130 (MS)				
bladder	ENSMUSP00000025773 (PM)	DPD4_MOUSE DNA polymerase delta subunit 4	nucleobase, nucleoside, nucleotide and nucleic acid metabolism,cell cycle,cell growth and/or maintenance,	transferase activity,	nucleus,
bladder	ENSMUSP00000047720 (PM)				
bladder	ENSMUSP00000071235 (MS)	PHX4_MOUSE Putative per-hexamer repeat protein 4			integral to membrane,
brain	ENSMUSP00000018595 (MS)				
brain	ENSMUSP00000026466 (MS)	TKNK_MOUSE Neurokinin B [Precursor]	signal transduction,cell surface receptor linked signal transduction,tachykinin signaling pathway,neuropeptide signaling pathway,cell-cell signaling,transmission of nerve impulse,neurophysiological process,organismal movement,neuromuscular	receptor binding,	

			physiological process,		
brain	ENSMUSP00000031268 (PM)			hydrolase activity,	
brain	ENSMUSP00000033198 (PM)	CRYM_MOUSE Mu-crystallin homolog	morphogenesis,organogenesis,	structural constituent of eye lens,	cytoplasm,
brain	ENSMUSP00000034023 (MS)	SRG1_MOUSE Scrapie-responsive protein 1 [Precursor]			
brain	ENSMUSP00000039418 (MS)	VHL_MOUSE Von Hippel-Lindau disease tumor suppressor	cell cycle,cell growth and/or maintenance,protein ubiquitination,macromolecule metabolism,	protein binding,	nucleus,
brain	ENSMUSP00000040342 (MS)		peptide hormone processing,hormone metabolism,macromolecule metabolism,	enzyme inhibitor activity,	cytoplasm,
brain	ENSMUSP00000046012 (MS)	APEL_MOUSE Apelin [Precursor]	signal transduction,cell surface receptor linked signal transduction,	receptor binding,	
brain	ENSMUSP00000065423 (PM)			carbohydrate binding,	
brain	ENSMUSP00000067770 (MS)				
brain	ENSMUSP00000075395 (PM)		nucleobase, nucleoside, nucleotide and nucleic acid metabolism,regulation of transcription, DNA-dependent,regulation of transcription from Pol II promoter,transcription from Pol II promoter,positive regulation of metabolism,regulation of nucleobase,regulation of metabolism,positive regulation of transcription,positive regulation of nucleobase,positive regulation of transcription from Pol II promoter,	transcription cofactor activity,protein binding,	nucleus,
cecum	ENSMUSP00000006692 (PM)	ER19_MOUSE Diphosphomevalonate decarboxylase	alcohol metabolism,lipid metabolism,cholesterol biosynthesis,phosphorus metabolism,phosphate metabolism,biosynthesis,macromolecule metabolism,	nucleotide binding,kinase activity,lyase activity,	
cecum	ENSMUSP00000025773 (PM)	DPD4_MOUSE DNA polymerase delta subunit 4	nucleobase, nucleoside, nucleotide and nucleic acid metabolism,cell cycle,cell growth and/or maintenance,	transferase activity,	nucleus,
cecum	ENSMUSP00000031146 (MS)	NEUU_MOUSE Neuromedin U-23 [Precursor]	cell motility,signal transduction,cell surface receptor linked signal transduction,neuropeptide signaling pathway,		
cecum	ENSMUSP00000031268 (PM)			hydrolase activity,	
cecum	ENSMUSP00000045737 (PM)	CND2_MOUSE Condensin complex subunit 2	M phase of mitotic cell cycle,nuclear division,cytokinesis,cell cycle,cell growth and/or maintenance,		nucleus,
cecum	ENSMUSP00000071235 (MS)	PHX4_MOUSE Putative per-hexamer repeat protein 4			integral to membrane,

cecum	ENSMUSP00000071936 (PM)	YV03_MOUSE Hypothetical protein PP2447 homolog			
distal colon	ENSMUSP00000031146 (MS)	NEUU_MOUSE Neuromedin U-23 [Precursor]	cell motility,signal transduction,cell surface receptor linked signal transduction,neuropeptide signaling pathway,		
proximal colon	ENSMUSP00000031146 (MS)	NEUU_MOUSE Neuromedin U-23 [Precursor]	cell motility,signal transduction,cell surface receptor linked signal transduction,neuropeptide signaling pathway,		
duodenum	ENSMUSP00000025704 (MS)		cytokinesis,cell growth and/or maintenance,		
duodenum	ENSMUSP00000031268 (PM)			hydrolase activity,	
duodenum	ENSMUSP00000039418 (MS)	VHL_MOUSE Von Hippel-Lindau disease tumor suppressor	cell cycle,cell growth and/or maintenance,protein ubiquitination,macromolecule metabolism,	protein binding,	nucleus,
duodenum	ENSMUSP00000045737 (PM)	CND2_MOUSE Condensin complex subunit 2	M phase of mitotic cell cycle,nuclear division,cytokinesis,cell cycle,cell growth and/or maintenance,		nucleus,
duodenum	ENSMUSP00000046012 (MS)	APEL_MOUSE Apelin [Precursor]	signal transduction,cell surface receptor linked signal transduction,	receptor binding,	
duodenum	ENSMUSP00000056752-D				
eye	ENSMUSP00000018595 (MS)				
eye	ENSMUSP00000026466 (MS)	TKNK_MOUSE Neurokinin B [Precursor]	signal transduction,cell surface receptor linked signal transduction,tachykinin signaling pathway,neuropeptide signaling pathway,cell-cell signaling,transmission of nerve impulse,neurophysiological process,organismal movement,neuromuscular physiological process,	receptor binding,	
eye	ENSMUSP00000031268 (PM)			hydrolase activity,	
eye	ENSMUSP00000033198 (PM)	CRYM_MOUSE Mu-crystallin homolog	morphogenesis,organogenesis,	structural constituent of eye lens,	cytoplasm,
eye	ENSMUSP00000036541 (PM)				
eye	ENSMUSP00000040342 (MS)		peptide hormone processing,hormone metabolism,macromolecule metabolism,	enzyme inhibitor activity,	cytoplasm,
eye	ENSMUSP00000063590 (PM)	N107_MOUSE Nuclear pore complex protein Nup107	cell growth and/or maintenance,		nucleus,endomembrane system,integral to membrane,
eye	ENSMUSP00000065836 (PM)				
eye	ENSMUSP00000067770 (MS)				
gall bladder	ENSMUSP00000016031 (PM)	HUTH_MOUSE Histidine	organic acid metabolism,amino acid and	lyase activity,ligase	cytoplasm,

		ammonia-lyase	derivative metabolism,amino acid metabolism,histidine metabolism,catabolism,biosynthesis,histidine family amino acid catabolism,amine metabolism,amine catabolism,	activity,	
gall bladder	ENSMUSP00000022722 (PM)	IRG1_MOUSE Immune-responsive protein 1 [Fragment]			
gall bladder	ENSMUSP00000023519 (PM)	HGD_MOUSE Homogentisate 1,2-dioxygenase	organic acid metabolism,amino acid and derivative metabolism,amino acid metabolism,aromatic compound metabolism,catabolism,aromatic amino acid family catabolism,amine metabolism,amine catabolism,aromatic compound catabolism,	oxidoreductase activity,	
gall bladder	ENSMUSP00000023832 (PM)	SM30_MOUSE Regucalcin		ion binding,	nucleus,cytoplasm,
gall bladder	ENSMUSP00000025249 (MS)	APOM_MOUSE Apolipoprotein M	cell growth and/or maintenance,	lipid transporter activity,	
gall bladder	ENSMUSP00000025773 (PM)	DPD4_MOUSE DNA polymerase delta subunit 4	nucleobase, nucleoside, nucleotide and nucleic acid metabolism,cell cycle,cell growth and/or maintenance,	transferase activity,	nucleus,
gall bladder	ENSMUSP00000029645 (PM)	T23O_MOUSE Tryptophan 2,3-dioxygenase	organic acid metabolism,amino acid and derivative metabolism,amino acid metabolism,tryptophan metabolism,biogenic amine metabolism,aromatic compound metabolism,amine metabolism,indole and derivative metabolism,heterocycle metabolism,	oxidoreductase activity,	
gall bladder	ENSMUSP00000032141 (PM)				
gall bladder	ENSMUSP00000034539 (PM)		nucleobase, nucleoside, nucleotide and nucleic acid metabolism,catabolism,macromolecule catabolism,macromolecule metabolism,	hydrolase activity,	nucleus,
gall bladder	ENSMUSP00000047720 (PM)				
gall bladder	ENSMUSP00000071235 (MS)	PHX4_MOUSE Putative per-hexamer repeat protein 4			integral to membrane,
gall bladder	ENSMUSP00000077192 (PM)		lipid metabolism,phospholipid metabolism,phosphatidylcholine biosynthesis,phospholipid biosynthesis,biosynthesis,macromolecule metabolism,membrane lipid biosynthesis,phosphatidylch	transferase activity,	cytoplasm, mitochondrial membrane,endomembrane system,integral to membrane,

			oline metabolism,glycerophospholipid biosynthesis,		
heart	ENSMUSP00000025554 (MS)	UTER_MOUSE Uteroglobin [Precursor]		enzyme inhibitor activity,steroid binding,	
ileum	ENSMUSP00000001667 (MS)	CASK_MOUSE Kappa-casein [Precursor]			
ileum	ENSMUSP00000006692 (PM)	ER19_MOUSE Kappa-casein [Precursor]	alcohol metabolism,lipid metabolism,cholesterol biosynthesis,phosphorus metabolism,phosphate metabolism,biosynthesis,macromolecule metabolism,	nucleotide binding,kinase activity,lyase activity,	
ileum	ENSMUSP00000025704 (MS)		cytokinesis,cell growth and/or maintenance,		
ileum	ENSMUSP00000025773 (PM)	DPD4_MOUSE DNA polymerase delta subunit 4	nucleobase, nucleoside, nucleotide and nucleic acid metabolism,cell cycle,cell growth and/or maintenance,	transferase activity,	nucleus,
ileum	ENSMUSP00000031268 (PM)			hydrolase activity,	
ileum	ENSMUSP00000034359 (MS)				
ileum	ENSMUSP00000034539 (PM)		nucleobase, nucleoside, nucleotide and nucleic acid metabolism,catabolism,macromolecule catabolism,macromolecule metabolism,	hydrolase activity,	nucleus,
ileum	ENSMUSP00000037484 (MS)			ion binding,	
ileum	ENSMUSP00000045737 (PM)	CND2_MOUSE Condensin complex subunit 2	M phase of mitotic cell cycle,nuclear division,cytokinesis,cell cycle,cell growth and/or maintenance,		nucleus,
ileum	ENSMUSP00000046012 (MS)	APEL_MOUSE Apelin [Precursor]	signal transduction,cell surface receptor linked signal transduction,	receptor binding,	
ileum	ENSMUSP00000071936 (PM)	YV03_MOUSE Hypothetical protein PP2447 homolog			
jejunum	ENSMUSP00000025704 (MS)		cytokinesis,cell growth and/or maintenance,		
jejunum	ENSMUSP00000031268 (PM)			hydrolase activity,	
jejunum	ENSMUSP00000034359 (MS)				
jejunum	ENSMUSP00000045737 (PM)	CND2_MOUSE Condensin complex subunit 2	M phase of mitotic cell cycle,nuclear division,cytokinesis,cell cycle,cell growth and/or maintenance,		nucleus,
jejunum	ENSMUSP00000046012 (MS)	APEL_MOUSE Apelin [Precursor]	signal transduction,cell surface receptor linked signal transduction,	receptor binding,	
kidney	ENSMUSP00000006692 (PM)	ER19_MOUSE Kappa-casein [Precursor]	alcohol metabolism,lipid metabolism,cholesterol biosynthesis,phosphorus metabolism,phosphate metabolism,biosynthesis,macromolecule metabolism,	nucleotide binding,kinase activity,lyase activity,	
kidney	ENSMUSP00000023282 (PM)	MIOX_MOUSE Inositol oxygenase	monosaccharide metabolism,alcohol metabolism,regulation of cell volume,signal	oxidoreductase activity,	cytoplasm,inclusion body,

			transduction,intracellular signaling cascade,cell growth and/or maintenance,catabolism,cellular osmoregulation,membrane organization and biogenesis,carbohydrate catabolism,osmoregulation, myo-inositol catabolism,hexose catabolism,cell homeostasis,macromolecule metabolism,alcohol catabolism,monosaccharide catabolism,inositol phosphate (MS)ediated signaling,		
kidney	ENSMUSP00000023519 (PM)	HGD_MOUSE Homogentisate 1,2-dioxygenase	organic acid metabolism,amino acid and derivative metabolism,amino acid metabolism,aromatic compound metabolism,catabolism,aromatic amino acid family catabolism,amine metabolism,amine catabolism,aromatic compound catabolism,	oxidoreductase activity,	
kidney	ENSMUSP00000025249 (MS)	APOM_MOUSE Apolipoprotein M	cell growth and/or maintenance,	lipid transporter activity,	
kidney	ENSMUSP00000075395 (PM)		nucleobase, nucleoside, nucleotide and nucleic acid metabolism,regulation of transcription, DNA-dependent,regulation of transcription from Pol II promoter,transcription from Pol II promoter,positive regulation of metabolism,regulation of nucleobase,regulation of metabolism,positive regulation of transcription,positive regulation of nucleobase,positive regulation of transcription from Pol II promoter,	transcription cofactor activity,protein binding,	nucleus,
liver	ENSMUSP00000016031 (PM)	HUTH_MOUSE Histidine ammonia-lyase	organic acid metabolism,amino acid and derivative metabolism,amino acid metabolism,histidine metabolism,catabolism,biosynthesis,histidine family amino acid catabolism,amine metabolism,amine catabolism,	lyase activity,ligase activity,	cytoplasm,
liver	ENSMUSP00000023519 (PM)	HGD_MOUSE Homogentisate 1,2-dioxygenase	organic acid metabolism,amino acid and derivative metabolism,amino acid metabolism,aromatic compound metabolism,catabolism,aromatic amino acid family catabolism,amine	oxidoreductase activity,	

			metabolism,amine catabolism,aromatic compound catabolism,		
liver	ENSMUSP00000023832 (PM)	SM30_MOUSE Regucalcin		ion binding,	nucleus,cytoplasm,
liver	ENSMUSP00000025249 (MS)	APOM_MOUSE Apolipoprotein M	cell growth and/or maintenance,	lipid transporter activity,	
liver	ENSMUSP00000029645 (PM)	T23O_MOUSE Tryptophan 2,3-dioxygenase	organic acid metabolism,amino acid and derivative metabolism,amino acid metabolism,tryptophan metabolism,biogenic amine metabolism,aromatic compound metabolism,amine metabolism,indole and derivative metabolism,heterocycle metabolism,	oxidoreductase activity,	
liver	ENSMUSP00000060495 (MS)	LCT2_MOUSE Leukocyte cell-derived chemotaxin 2 [Precursor]	sensory perception,response to external stimulus,neurophysiological process,		
liver	ENSMUSP00000077192 (PM)		lipid metabolism,phospholipid metabolism,phosphatidylcholine biosynthesis,phospholipid biosynthesis,biosynthesis,macromolecule metabolism,membrane lipid biosynthesis,phosphatidylcholine metabolism,glycerophospholipid biosynthesis,	transferase activity,	cytoplasm, mitochondrial membrane,endomembrane system,integral to membrane,
lung	ENSMUSP00000025554 (MS)	UTER_MOUSE Uteroglobin [Precursor]		enzyme inhibitor activity,steroid binding,	
lung	ENSMUSP00000044493 (MS)				
lung	ENSMUSP00000044862-D				
lung	ENSMUSP00000071235 (MS)	PHX4_MOUSE Putative per-hexamer repeat protein 4			integral to membrane,
muscle	ENSMUSP00000030705 (PM)	RFA2_MOUSE Replication protein A 32 kDa subunit	nucleobase, nucleoside, nucleotide and nucleic acid metabolism,cell cycle,cell growth and/or maintenance,	nucleic acid binding,	nucleus,
muscle	ENSMUSP00000065423 (PM)			carbohydrate binding,	
spleen	ENSMUSP00000025773 (PM)	DPD4_MOUSE DNA polymerase delta subunit 4	nucleobase, nucleoside, nucleotide and nucleic acid metabolism,cell cycle,cell growth and/or maintenance,	transferase activity,	nucleus,
spleen	ENSMUSP00000027162 (MS)		response to biotic stimulus,	antigen binding,receptor binding,peptide binding,	plasma membrane,integral to membrane,
spleen	ENSMUSP00000030180 (MS)		cell activation,immune response,signal transduction,response to biotic stimulus,T-cell activation,immune cell activation,lymphocyte activation,regulation of T-	receptor signaling protein activity,protein binding,	plasma membrane,integral to membrane,

			cell activation,regulation of cell activation,		
spleen	ENSMUSP00000030705 (PM)	RFA2_MOUSE Replication protein A 32 kDa subunit	nucleobase, nucleoside, nucleotide and nucleic acid metabolism,cell cycle,cell growth and/or maintenance,	nucleic acid binding,	nucleus,
spleen	ENSMUSP00000032141 (PM)				
spleen	ENSMUSP00000034539 (PM)		nucleobase, nucleoside, nucleotide and nucleic acid metabolism,catabolism,macromolecule catabolism,macromolecule metabolism,	hydrolase activity,	nucleus,
spleen	ENSMUSP00000044493 (MS)				
spleen	ENSMUSP00000047720 (PM)				
spleen	ENSMUSP00000063590 (PM)	N107_MOUSE Nuclear pore complex protein Nup107	cell growth and/or maintenance,		nucleus,endomembrane system,integral to membrane,
spleen	ENSMUSP00000071235 (MS)	PHX4_MOUSE Putative per-hexamer repeat protein 4			integral to membrane,
spleen	ENSMUSP00000078413-D	FBS1_MOUSE Fibrosin-1 [Fragment]		receptor binding,	
testis	ENSMUSP00000020990 (MS)	COLI_MOUSE Corticotropin-lipotropin [Precursor]	signal transduction,cell surface receptor linked signal transduction,neuropeptide signaling pathway,	receptor binding,	
testis	ENSMUSP00000022019 (MS)	IL9_MOUSE Interleukin-9 [Precursor]	immune response,response to biotic stimulus,	receptor binding,	
testis	ENSMUSP00000025704 (MS)		cytokinesis,cell growth and/or maintenance,		
testis	ENSMUSP00000031268 (PM)			hydrolase activity,	
testis	ENSMUSP00000031876 (MS)				
testis	ENSMUSP00000032481 (MS)		reproduction,gametogenesis, spermatid development,sexual reproduction,cell differentiation,	nucleic acid binding,protein binding,enzyme activator activity,	nucleus,cytoplasm,
testis	ENSMUSP00000033691 (MS)	TSX_MOUSE Testis-specific protein TSX			
testis	ENSMUSP00000037484 (MS)			ion binding,	
testis	ENSMUSP00000039418 (MS)	VHL_MOUSE Von Hippel-Lindau disease tumor suppressor	cell cycle,cell growth and/or maintenance,protein ubiquitination,macromolecule metabolism,	protein binding,	nucleus,
testis	ENSMUSP00000044862-D				
testis	ENSMUSP00000045737 (PM)	CND2_MOUSE Condensin complex subunit 2	M phase of mitotic cell cycle,nuclear division,cytokinesis,cell cycle,cell growth and/or maintenance,		nucleus,
testis	ENSMUSP00000045925 (MS)D		macromolecule metabolism,	protein binding,	ubiquitin ligase complex,cyt

					oplasm,
testis	ENSMUSP00000047588 (MS)			protein binding,	nucleus,
testis	ENSMUSP00000059448 (MS)	TUR8_MOUSE Tumor rejection antigen P815A			nucleus,integral to membrane,
testis	ENSMUSP00000059630 (MS)	HSP3_MOUSE Sperm protamine P3	reproduction,M phase of mitotic cell cycle,nuclear division,cell cycle,gametogenesis,cell growth and/or maintenance,sexual reproduction,	nucleic acid binding,	nucleus,chromosome,
testis	ENSMUSP00000063590 (PM)	N107_MOUSE Nuclear pore complex protein Nup107	cell growth and/or maintenance,		nucleus,endomembrane system,integral to membrane,
testis	ENSMUSP00000065423 (PM)			carbohydrate binding,	
testis	ENSMUSP00000065836 (PM)				
testis	ENSMUSP00000067770 (MS)				
testis	ENSMUSP00000077192 (PM)		lipid metabolism,phospholipid metabolism,phosphatidylcholine biosynthesis,phospholipid biosynthesis,biosynthesis,macromolecule metabolism,membrane lipid biosynthesis,phosphatidylcholine metabolism,glycerophospholipid biosynthesis,	transferase activity,	cytoplasm, mitochondrial membrane,endomembrane system,integral to membrane,
testis	ENSMUSP00000078413-D	FBS1_MOUSE Fibrosin-1 [Fragment]		receptor binding,	
thymus	ENSMUSP00000000028 (PM)	CC45_MOUSE CDC45-related protein	nucleobase, nucleoside, nucleotide and nucleic acid metabolism,cell cycle,cell growth and/or maintenance,		nucleus,
thymus	ENSMUSP00000020765 (PM)	DPD2_MOUSE DNA polymerase delta subunit 2	nucleobase, nucleoside, nucleotide and nucleic acid metabolism,cell cycle,cell growth and/or maintenance,	transferase activity,	nucleus,
thymus	ENSMUSP00000021130 (MS)				
thymus	ENSMUSP00000021164 (MS)				
thymus	ENSMUSP00000025704 (MS)		cytokinesis,cell growth and/or maintenance,		
thymus	ENSMUSP00000025773 (PM)	DPD4_MOUSE DNA polymerase delta subunit 4	nucleobase, nucleoside, nucleotide and nucleic acid metabolism,cell cycle,cell growth and/or maintenance,	transferase activity,	nucleus,
thymus	ENSMUSP00000027162 (MS)		response to biotic stimulus,	antigen binding,receptor binding,peptide binding,	plasma membrane,integral to membrane,
thymus	ENSMUSP00000030180 (MS)		cell activation,immune response,signal transduction,response to biotic stimulus,T-cell activation,immune cell activation,lymphocyte activation,regulation of T-cell activation,regulation of	receptor signaling protein activity,protein binding,	plasma membrane,integral to membrane,

			cell activation,		
thymus	ENSMUSP00000030705 (PM)	RFA2_MOUSE Replication protein A 32 kDa subunit	nucleobase, nucleoside, nucleotide and nucleic acid metabolism,cell cycle,cell growth and/or maintenance,	nucleic acid binding,	nucleus,
thymus	ENSMUSP00000034539 (PM)		nucleobase, nucleoside, nucleotide and nucleic acid metabolism,catabolism,macromolecule catabolism,macromolecule metabolism,	hydrolase activity,	nucleus,
thymus	ENSMUSP00000038204 (MS)	RAG2_MOUSE V(D)J recombination-activating protein 2	cell activation,nucleobase, nucleoside, nucleotide and nucleic acid metabolism,immune response,response to biotic stimulus,morphogenesis,organogenesis,lymphocyte differentiation,cell differentiation,immune cell activation,lymphocyte activation,	nucleic acid binding,peroxidase activity,oxidoreductase activity,hydrolase activity,	nucleus,
thymus	ENSMUSP00000044493 (MS)				
thymus	ENSMUSP00000045737 (PM)	CND2_MOUSE Condensin complex subunit 2	M phase of mitotic cell cycle,nuclear division,cytokinesis,cell cycle,cell growth and/or maintenance,		nucleus,
thymus	ENSMUSP00000047720 (PM)				
thymus	ENSMUSP00000063590 (PM)	N107_MOUSE Nuclear pore complex protein Nup107	cell growth and/or maintenance,		nucleus,endomembrane system,integral to membrane,

*PM – pre-metazoan protein; MS – metazoan-specific protein

Supplementary Table 3. Tissue list of the main dataset. Cluster representatives are indicated using bold face.

Tissue	Cluster ID
**Antrum**	1
**Appendix**	2
Bladder	3
Gall bladder	3
**Lung**	3
**Brain**	4
Eye	4
Cecum	5
**Duodenum**	5
Ileum	5
Jejunum	5
**Distal colon**	6
Proximal colon	6
**Heart**	7
**Kidney**	8
**Liver**	9
**Muscle**	10
Spleen	11
**Thymus**	11
**Testis**	12
Testis	12
**Vas deferens**	13

Supplementary Table 4. Tissue list of the additional dataset. Cluster representatives are indicated using bold face.

Tissue	Cluster ID
Amygdala	1
**Cerebellum**	1
Cerebral cortex	1
Dorsal root ganglion	1
Dorsal striatum	1
Frontal cortex	1
Hippocampus	1
Hypothalamus	1
Olfactory bulb	1
Preoptic	1
Retina	1
Lower spinal cord	1
Upper spinal cord	1
Substantianigra	1
Trigeminal	1
b220.bcell	2
cd4.Tcell	2
cd8.Tcell	2
Lymph node	2
**Thymus**	2
**Heart**	3
**Testis**	4
Adipose tissue	5
Adrenal gland	5
Bladder	5
**Lung**	5
Prostate	5
**Kidney**	6
**Liver**	7
**Salivary gland**	8
Digits	9
**Epidermis**	9
Snout epidermis	9
Tongue epidermis	9
**Bone**	10
**Large intestine**	11
Small intestine	11
**Spleen**	12
**Pituitary**	13
Medial olfactory epithelium (MOE)	14
**Vomeral-nasal organ (VMO)**	14
**Thyroid**	15
**Stomach**	16
**Pancreas**	17

**Brown fat**	18
**Skeletal muscle**	19
**Bone marrow**	20
